# Fully-automated production of [^68^Ga]Ga-Trivehexin for clinical application and its biodistribution in healthy volunteers

**DOI:** 10.3389/fonc.2024.1445415

**Published:** 2024-08-02

**Authors:** Binchen Wang, Yaqun Jiang, Jiaxu Zhu, Huiqin Wu, Jianyuan Wu, Ling Li, Jianying Huang, Zhiwei Xiao, Yong He

**Affiliations:** ^1^ Department of Nuclear Medicine, Zhongnan Hospital of Wuhan University, Wuhan, China; ^2^ Clinical Trial Center, Zhongnan Hospital of Wuhan University, Wuhan, China

**Keywords:** fully-automated, Trivehexin, α_v_β_6_-integrin, PET, biodistribution

## Abstract

**Background:**

The α_v_β_6_-integrin targeting trimeric ligand [^68^Ga]Ga-Trivehexin has emerged as a promising candidate for clinical application due to its clinical imaging potentials in various malignant cancers. Our objective was to develop a simplified and reproducible module-based automated synthesis protocol to expand its availability in clinical application.

**Methods:**

The pH value and the precursor load of radiolabeling were explored using an iQS-TS fully-automated module. Radiochemical purity was evaluated by radio-HPLC and radio-TLC. The ethanol content, radionuclide purity and identity, bacterial endotoxins, sterility, and stability of the final product [^68^Ga]Ga-Trivehexin were all tested. Biodistribution of [^68^Ga]Ga-Trivehexin in healthy volunteers was also conducted.

**Results:**

The synthesis was explored and established using fully-automated module with outstanding radiochemical purity (>99%). Considering molar activity and economic costs, a pH of 3.6 and precursor dose of 30 μg were determined to be optimal. All relevant quality control parameters were tested and met the requirement of European Pharmacopoeia. *In vitro* stability test and imaging in healthy volunteer indicated the practical significance in clinical routines.

**Conclusions:**

A fully-automated synthesis protocol for [^68^Ga]Ga-Trivehexin using the iQS-TS synthesis module was achieved and conformed to the clinical quality standards.

**Clinical trial registration:**

ClinicalTrials.gov, NCT05835570. Registered 28 April 2023, https://www.clinicaltrials.gov/study/NCT05835570.

## Introduction

Integrin α_v_β_6_, one of a class of 24 transmembrane cell adhesion receptors, is exclusively expressed by epithelial cells and relevant to the activation of transforming growth factor β (TGF-β), a powerful growth-inhibiting factor which regulates gene transcription, DNA replication, and cell proliferation through Smad-dependent signaling pathways (1). Currently, α_v_β_6_ is indicated to be closely associated with carcinogenesis (2). By binding to an Arg-Gly-Asp (RGD) sequence of latency-associated peptide (LAP), α_v_β_6_-integrin could promote releasing TGF-β into the extracellular space. However, due to the loss of certain downstream signaling components, including p53 (3) or Smad4 (4), tumor cells become resistant to the growth inhibition by TGF-β which in turn, makes high levels of TGF-β more conductive to the tumor growth (5). High expression of α_v_β_6_ has been reported to be prevalent in pancreatic ductal adeno carcinoma (PDAC), as well as in other carcinomas, and most importantly, squamous cells (6), basal cells, lung adenocarcinoma, and colon (7). Consequently, α_v_β_6_-integrin is considered as a valuable target for molecular imaging to accurately delineate tumor margins or assess invasiveness, as well as therapeutic interventions with specific radioligands. Among these α_v_β_6_ targeted ligands (8–11), cyclic peptides showed promising clinical potentials, in which the trimeric ligand [^68^Ga]Ga-Trivehexin, reported by Johannes Notni et al. (**Supplementary Figure S1**) (12) revealed improved and more suitable  tumor visualization capacity in comparison to those monomeric peptides.

To our knowledge, despite the robust manual in-house kit-like synthesis protocol of [^68^Ga]Ga-Trivehexin reported by Thakral et al. (13), no detailed optimization and guidance of a fully-automated radiosynthesis protocol were available so far. In this study, we aimed to establish a fully-automated radiosynthesis approach of [^68^Ga]Ga-Trivehexin with module iQS-Theranostics Synthesizer (iQS-TS). Detailed evaluations of the synthetic efficacy, quality control, *in vivo* biodistribution, and dosimetry of [^68^Ga]Ga-Trivehexin in healthy volunteers were validated to demonstrate the feasibility, reproducibility and simplified clinical application of this procedure.

## Materials and methods

### Materials and reagents

Trivehexin was purchased from CSBio Co with chemical purity greater than 95% (1 mg/pack, 20 Kelly Court, Menlo Park, CA, 94025 USA), and kept as aqueous aliquots of 1 mg/mL at -28°C (12). Gradient grade solvents for radio high-performance-liquid-chromatography (radio-HPLC) system and absolute ethanol for formulation were purchased from Sigma-Aldrich. Ultrapure hydrochloric acid for elution was obtained from Merck Millipore. Sodium acetate was purchased from Sigma-Aldrich. Ultrapure water was used in all experiments. The pH value was measured using the Mettler Toledo S210-K pH meter.

The ^68^Ge/^68^Ga generator (Isotope Technologies Garching GmbH, Garching, Germany) and iQS-TS synthesis module (Isotope Technologies Garching GmbH, Garching, Germany) were used. All commercially available sterile, single-use cassettes (produced according to GMP) for radio synthesis were purchased from Isotope Technologies Garching GmbH. Sterile vacuum vial from Huayi Isotope Co. and Millipore Express membrane filter unit (0.22 μm) from Merck Millipore were used for formulation. A borehole counter (Capintec, Inc., USA) was used for activity counting. Radiochemical purity and yield were tested using radio-HPLC (Waters Corporation, USA) equipped with a 1525 Binary Pump, a 2489 UV/visible detector, an FC-3200 flow count radiation detector (Eckert & Ziegler, Germany), and a 4.6 × 250mm Luna C18 HPLC column (Phenomenex, CA, USA). The instant thin-layer chromatography-silica gel (iTLC-SG) scanner (Hefei Zhongcheng Electromechanical Technology Development Co., LTD) was used.

### Synthesis preparation

Trivehexin was prepared in 10-50 μg (2.3×10^-9^ to 1.15×10^-8^ mol) aliquots (0.8 μg/μL) with ultrapure water. Sodium acetate solutions with different concentrations (0.13, 0.15, 0.16 0.18, 0.21 and 0.22 M) were prepared for pH adjustment. Hydrochloric acid solution (0.05 M) was prepared from 30% HCl and ultrapure water. A low bioburden cassette and a reagent set were included for single use.

### Automatically radiosynthesis of [^68^Ga]Ga-Trivehexin

Fully automated radiosynthesis of [^68^Ga]Ga-Trivehexin was conducted on the iQS-TS module using a disposable cassette equipped with a C18 cartridge, ethanol, and 0.9% saline solution. Detailed configuration is presented in **Supplementary Figure S2**. The reaction vial pre-loaded with a mixture of precursor and 1 mL NaOAc aqueous solution, was manually connected to the module and ^68^Ge/^68^Ga radionuclide generator, and then pre-heated to 95°C. Remaining steps were automatically completed by the module. It was recommended to use 4.15 mL 0.05M HCl as the elution medium in HCl vial to ensure sufficient elution volume of 4 mL, and [^68^Ga]GaCl_3_ solution was eluted with 4.0 mL 0.05 M HCl solution at a flow rate of 4 mL/min. The C18 cartridge was pre-conditioned with 70% aqueous Ethanol solution and 0.9% saline. After the formulation, [^68^Ga]Ga-Trivehexin was obtained as a 9 mL 0.9% saline solution with Ethanol content less than 5%. The detailed steps were in **Supplementary Table S1**.

To assess the impact of the pH value and amount of precursor, pH values of 2.0, 2.4, 2.9, 3.6, 4.0, 4.5, and precursor loads of 10, 20, 30, 40, 50 μg were evaluated respectively. Radioactivity of segments, including the product vial, reaction vial, C18 cartridge, waste vial, and the sterile filter membrane were detected immediately after the synthesis to give a detailed radioactivity distribution of ^68^Ga on the cassette.

Each condition was repeated for three times respectively to confirm the reproducibility of the radiosynthesis method.

### Quality control

Radiochemical purity was evaluated by radio-HPLC and radio-TLC. For radio-HPLC, a mobile phase of H_2_O with 0.1% trifluoroacetic acid (TFA) (*v*/*v*, phase A) and acetonitrile with 0.1% TFA (*v*/*v*, phase B) was used with a flow rate of 1.0 mL/min and a linear elution gradient as follows: 0-5 min (0-95% A); 5-10 min (95-30% A);10-20 min (30-0% A). For radio-TLC, 0.4 M ammonium acetate solution/methanol (*v*/*v*, 1/1) (A) and 0.1 M citrate buffer (B) were used as mobile phases, respectively.

The ethanol content was determined by gas chromatography (GC).

The radionuclide purity and identity were determined by quantifying the ^68^Ge breakthrough and measuring the half-life. To quantify the ^68^Ge breakthrough, the final product (no less than 1 × 10^7^ Bq) was diluted to 1 mL with pure water and decayed to less than 5 × 10^6^ Bq. Radiation was determined and analysis immediately (A_0_) and 48 hours later (A_1_) with a gamma spectrometry. Subsequently, ^68^Ge breakthrough was determined with the following equation. A_1_ and A_0_ as the radioactivity of different time points.


$${\;}^{68}\text{Ge breakthrough}=\frac{{\text{A}}_{1}}{{\text{A}}_{0}}$$

To calculate the half-life (*T*_1/2_), the radioactivity of [^68^Ga]Ga-Trivehexin was accurately measured and recorded 5 times every 5 minutes. The half-life was calculated based on the equations below, with λ as the decay constant, k as the slope, N_0_ and N as the radioactivity, t_0_ and t as time point, *T*_1/2_ as half-life.


$$\text{λ}=\left|\text{k}\right|=\frac{{\text{lnN}}_{0}-\text{lnN}}{{\text{t}}_{0}-\text{t}}$$

$$T_{1/2}=\frac{ln2}{\lambda }$$

The pH value was measured by a pH meter.

The bacterial endotoxins and sterility were determined following the methods in the Pharmacopoeia of the People’s Republic of China 2020, number 1100 and 1143. The samples of the final product (0.2 mL each) were prepared after the total radioactive decay.

The stability of [^68^Ga]Ga-Trivehexin in formulation and serum was further evaluated in Phosphate Buffer Saline (PBS) and Fetal Bovine Serum (FBS). After co-incubation of the final product with PBS or FBS for 60 or 120 min, radio-HPLC was used for the analysis.

### PET imaging and dosimetry estimation in humans

The clinical study was approved by Medical Ethics Committee, Zhongnan Hospital of Wuhan University and registered at ClinicalTrials.gov (NCT05835570). PET/CT imaging was performed using a Siemens-Biograph mCT PET/CT scanner (Siemens Healthineers, Erlangen, Germany). No special preparation was needed for [^68^Ga]Ga-Trivehexin PET/CT. The injection dose was 87-122 MBq (70.7 MBq/mL, 90.9 MBq/nmol) and was determined on the basis of the patient’s weight (1.85 MBq/kg). PET/CT images were scanned from the top of the head to the upper thigh. PET data were obtained in a three-dimensional mode (matrix 200 × 200) of 6-8 bed positions (3 min/bed). Attenuation correction of PET images was performed using low-dose CT data. Image reconstruction was performed using the TrueX and time-of-flight (ultrahigh-definition PET) algorithms.

Applications of [^68^Ga]Ga-Trivehexin in 3 healthy volunteers were done with written informed consent, and details of the volunteers were shown in **Supplementary Table S2**. There were no adverse or clinically detectable pharmacologic effects and no significant changes in vital signs. The PET scan was performed at 10 min, 35 min, and 60 min after intravenous injection, without repeated CT scans. Another PET/CT imaging sequence was obtained at 150 min post-injection, as the subject had to leave the scanner for voiding. Based on human PET data, the dosimetry values were calculated using OLINDA V2.2 to calculate organ doses and effective dose (as defined by International Commission on Radiological Protection publication 103) (14) using the International Commission on Radiological Protection publication 89 (15) adult human male and female models.

## Results

### Optimal radiolabeling condition

**Figure 1:**
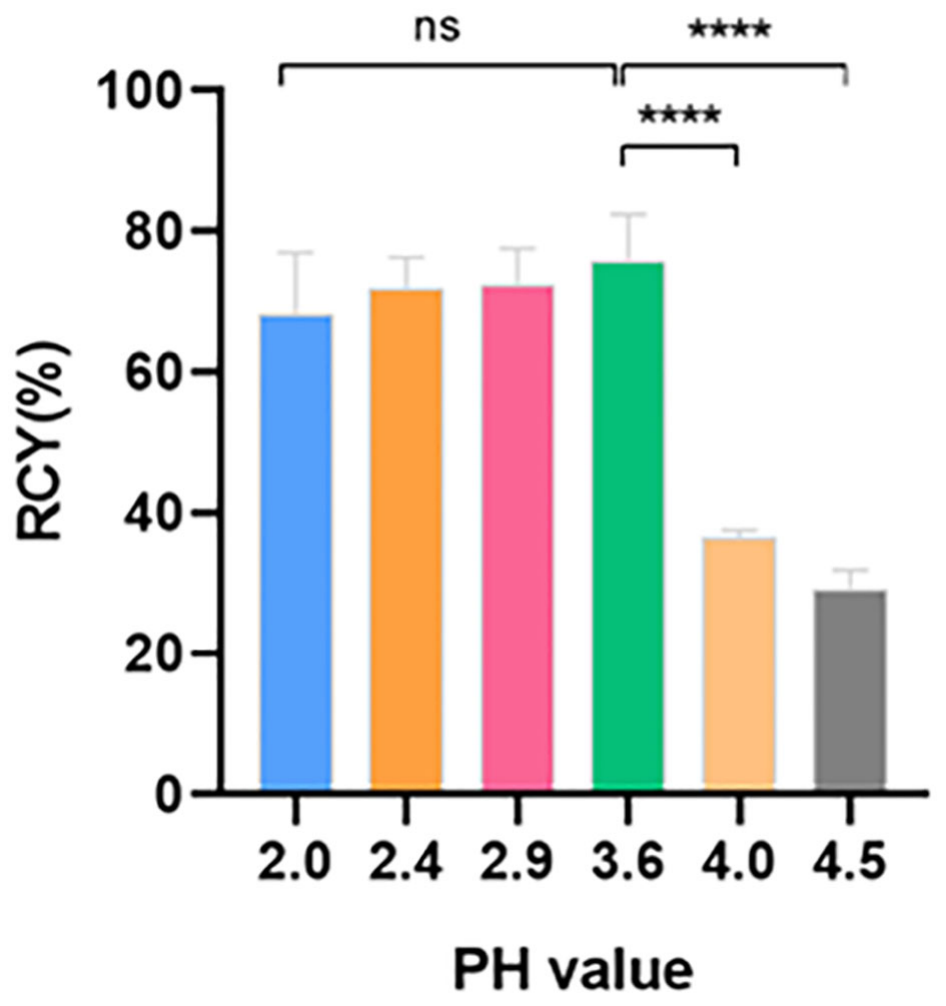
RCY at different pH values (n = 3). RCY, radiochemical yield; ns, not statistically significant; * *p* < 0.05.

To achieve a homogenous and stable temperature distribution in the reaction vial, 95°C and 10 min reaction time were keeping constant throughout the optimization. With 30 μg precursors, higher RCY of 72.4 ± 4.2% and 75.7 ± 5.4% appeared at pH 2.9 and 3.6, respectively (**Figure 1**). Increasing alkaline conditions resulted in continuous RCY decreases to 29.2 ± 1.8%, and significantly increased percentages on the C18 cartridge from 3.4 ± 1.8% to 47.2 ± 1.8%. On the contrary, acidity enhancement merely lowered the labeling efficacy with increased distributions observed in the waste (22.1 ± 6.1% and 21.2 ± 5.9%) (**Table 1**). No significant changes were found in the reaction vial and on the sterile filter membrane through all the pH changes.

**Table 1:** Activity ratio measured on different parts of cassette immediately after synthesis at different pH values (n = 3).

Reaction pH	Precursor load (μg)	Product vial (%)	Reaction vial (%)	C18 cartridge (%)	Waste (%)	Sterile filter membrane (%)	RCY (%)
2.0	30	73.1 ± 5.2	2.5 ± 0.1	3.3 ± 0.7	22.1 ± 6.1	2.0 ± 1.0	68.3 ± 7.1
2.4	30	69.7 ± 7.7	3.2 ± 0.4	4.5 ± 1.4	21.2 ± 5.9	1.5 ± 1.0	68.6 ± 6.7
2.9	30	76.6 ± 2.5	1.8 ± 0.4	6.5 ± 3.8	12.6 ± 3.2	2.5 ± 0.5	72.4 ± 4.2
3.6	30	76.7 ± 4.3	1.8 ± 0.1	3.4 ± 1.8	14.4 ± 2.2	3.7 ± 1.7	75.7 ± 5.4
4.0	30	35.1 ± 0.8	3.9 ± 1.0	45.0 ± 1.2	13.2 ± 0.9	2.8 ± 0.5	36.3 ± 1.0
4.5	30	29.9 ± 0.9	3.7 ± 0.4	47.2 ± 1.8	17.3 ± 1.6	1.9 ± 0.3	29.2 ± 1.8

**Figure 2:**
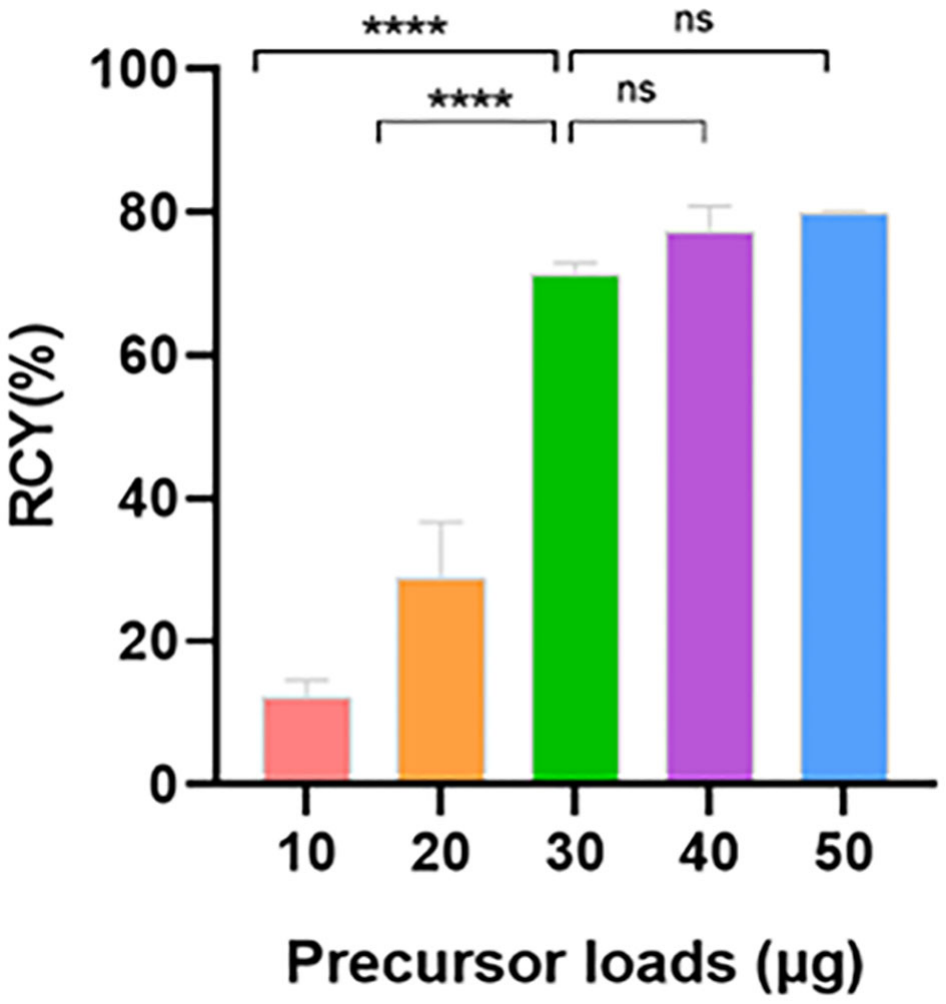
RCY for different precursor (n = 3). RCY, radiochemical yield; * *p* < 0.05.

With the optimized pH value, we next assessed the impact of precursor loads (**Table 2**). Notably, similar high RCYs of 75.7 ± 5.4%, 77.4 ± 2.8% and 79.8 ± 0.3% were observed with 30-50 μg precursor, respectively (**Figure 2**). The use of lower loads significantly reduced the RCY to 12.2 ± 2.0% with most activity remaining on the C18 cartridge and in the waste vial. Considering the economic cost and labeling efficacy, we identified that pH = 3.6 and precursor load of 30 μg (7×10^-6^ mmol) were the optimal radiolabeling condition for further quality control and clinical evaluation.

**Table 2:** Activity ratio measured on different parts of cassette immediately after synthesis with different precursor loads (n = 3).

Precursor load (μg)	Reaction pH	Product vial (%)	Reaction vial (%)	C18 cartridge (%)	Waste (%)	Sterile filter membrane (%)	RCY (%)
10	3.6	11.9 ± 2.0	5.6 ± 2.6	40.4 ± 18.3	41.0 ± 20.8	1.1 ± 0.3	12.2 ± 2.0
20	3.6	29.9 ± 7.8	5.7 ± 1.5	16.7 ± 10.8	45.1 ± 7.4	2.7 ± 1.7	28.8 ± 6.4
30	3.6	76.7 ± 4.3	1.8 ± 0.1	3.4 ± 1.8	14.4 ± 2.2	3.7 ± 1.7	75.7 ± 5.4
40	3.6	78.8 ± 0.5	4.9 ± 2.1	3.7 ± 1.3	9.5 ± 1.7	3.2 ± 0.8	77.4 ± 2.8
50	3.6	80.0 ± 5.4	3.8 ± 3.2	3.0 ± 0.8	10.8 ± 1.3	2.5 ± 0.9	79.8 ± 0.3

### Quality control of [^68^Ga]Ga-Trivehexin

The radiochemical purity was evaluated using both radio-HPLC and radio-TLC methods. With radio-HPLC, [^68^Ga]Ga-Trivehexin was detected with a purity of 99.79% at t_R_ = 10.01 min. Radioactive impurities were detected at t_R_ = 10.93 min, constituting approximate 0.21% of the total radioactivity. No significant amount of free gallium-68 was observed (**Supplementary Figure S3A**). UV-HPLC Chromatogram of [^68^Ga]Ga-Trivehexin was shown in **Supplementary Figure S3B**.

**Figure 3:**
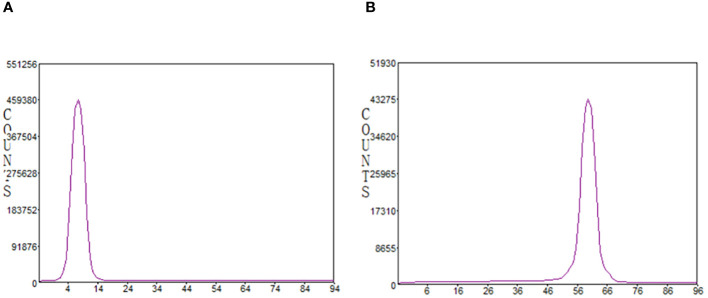
Radiochemical purity analyzed by radio-iTLC. **(A)** mobile phase: 0.4M ammonium acetate solution/methanol (1/1); **(B)** mobile phase: 0.1M citrate buffer.

In the radio-TLC analysis, for both mobile phases (ammonium acetate/methanol *v*/*v*=1/1 and citrate buffer), no free gallium-68 or ^68^Ga-colloide was observed. The product was detected at R_f_ = 0.0 and R_f_ = 1.0, respectively (**Figure 3**).

**Figure 4:**
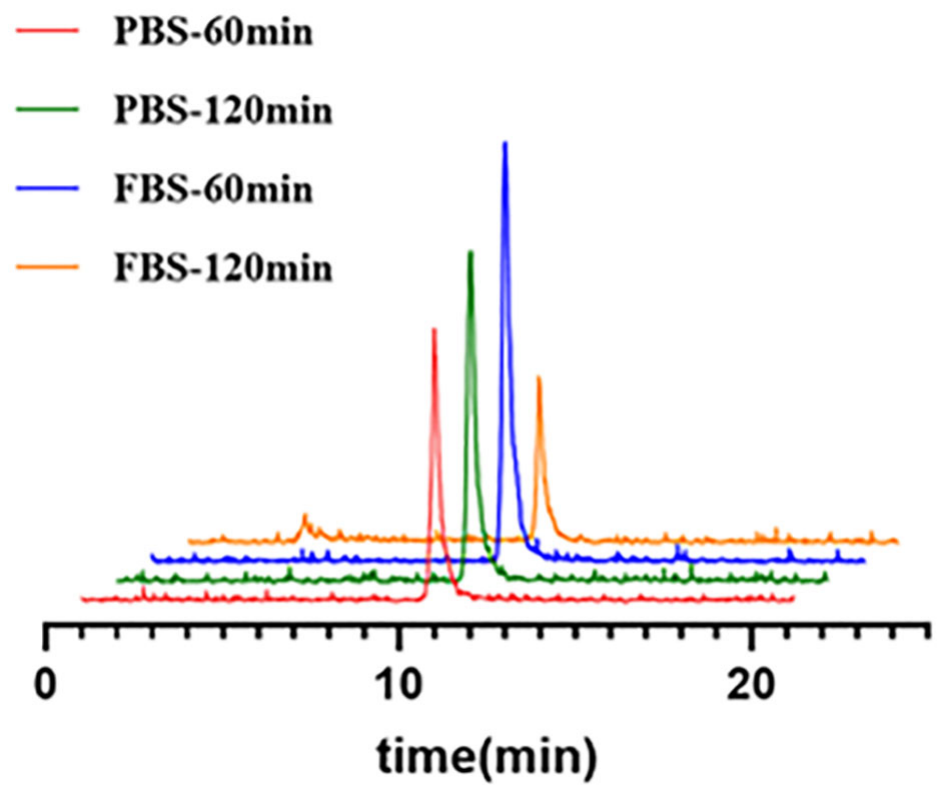
The stability of [^68^Ga]Ga-Trivehexin in both PBS and FBS. PBS, Phosphate Buffer Saline; FBS, Fetal Bovine Serum.

In summary, all the tested quality control parameters met the requirement of the European Pharmacopoeia and were shown in **Table 3** with the release criteria. Subsequent tests in PBS and FBS revealed excellent *in vitro* formulation and serum stability of [^68^Ga]Ga-Trivehexin with up to 98.9% and 74.2% of intact tracer remained after 2 h co-incubation, respectively (**Figure 4**).

**Table 3:** Summary of the product specifications for [^68^Ga]Ga-Trivehexin (n = 3).

Test	Criteria	Product (n = 3)
Visual inspection	Clear, colorless	Clear, colorless
Radio chemical purity (radio-HPLC)	>95%	>99%
Radio chemical purity (radio-iTLC)	>95%	>99%
Volume	2-10 mL	9 mL
Dose pH	5.0-8.0	5.8
Endotoxin analysis	<15EU/mL	Pass
Ethanol content	≤10%	<5%
Radionuclide identity	62-74min	67.6min
^68^Ge breakthrough	<0.001%	0.0004%
Sterility testing	No colony growth out to 14 days	Pass

### Biodistribution of [^68^Ga]Ga-Trivehexin and in-human dosimetry estimate

**Figure 5:**
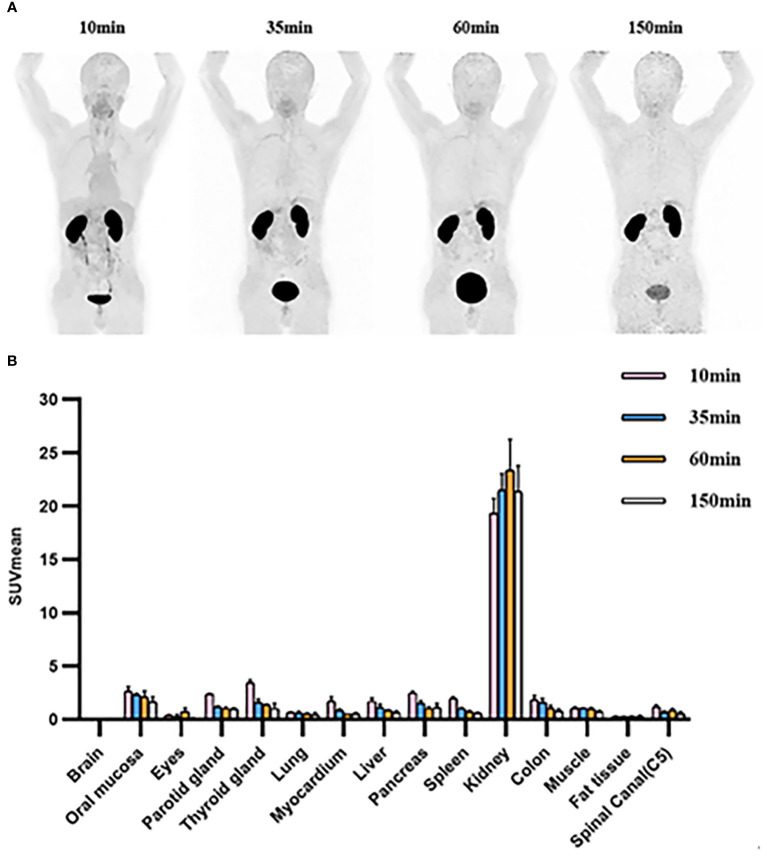
**(A)** [^68^Ga]Ga-Trivehexin PET of healthy volunteer #1, 122 MBq, anterior maximum intensity projections (MIPs) scaled to SUV 10. **(B)** SUVmean of organs at 10min, 35min, 60min, and 150 min after injection in healthy volunteers. SUV, Standardized uptake value. All the data are expressed as mean ± SD (n = 3).

In accordance with previously reported data (12), varying degrees of low diffuse physiological uptake were observed in major organs, with maximum uptake distributed in kidneys (**Table 4**, **Figure 5**). Further dosimetry analysis revealed the total body effective dose of 1.67E-02 mSv/MBq, with the highest effective dose in kidneys (2.26E-01 mSv/MBq) followed by urinary bladder wall (8.24E-02 mSv/MBq) and adrenals (3.22E-02 mSv/MBq). Dosimetry estimates were calculated with OLINDA V2.2, as shown in **Supplementary Table S3**.

**Table 4:** Standard uptake values (SUVmean ± SD) of [^68^Ga]Ga-Trivehexin in healthy volunteers (n = 3).

	15min p.i.	30min p.i.	60min p.i.	150min p.i.
**Brain**	0.04 ± 0.01	0.03 ± 0.01	0.01 ± 0.00^*^	0.01 ± 0.00^*^
**Oral mucosa**	2.60 ± 0.38	2.28 ± 0.13	2.12 ± 0.43	1.59 ± 0.42
**Eyes**	0.36 ± 0.04	0.28 ± 0.14	0.71 ± 0.28	0.12 ± 0.03
**Parotid gland**	2.31 ± 0.09	1.19 ± 0.06	1.01 ± 0.10	0.97 ± 0.07
**Thyroid gland**	3.41 ± 0.22	1.54 ± 0.29	1.37 ± 0.04	1.01 ± 0.37
**Lung**	0.65 ± 0.03	0.58 ± 0.14	0.53 ± 0.05	0.45 ± 0.13
**Myocardium**	1.69 ± 0.35	0.83 ± 0.13	0.52 ± 0.01	0.50 ± 0.09
**Liver**	1.68 ± 0.26	1.11 ± 0.23	0.82 ± 0.07	0.65 ± 0.10
**Pancreas**	2.46 ± 0.12	1.47 ± 0.24	1.01 ± 0.11	1.13 ± 0.28
**Spleen**	1.95 ± 0.12	1.01 ± 0.06	0.76 ± 0.01	0.55 ± 0.07
**Kidney**	19.32 ± 1.13	21.5 ± 1.28	23.35 ± 2.38	21.3 ± 2.00
**Colon**	1.86 ± 0.31	1.52 ± 0.36	1.07 ± 0.21	0.78 ± 0.16
**Muscle**	1.04 ± 0.10	1.04 ± 0.01	0.97 ± 0.10	0.68 ± 0.10
**Fat tissue**	0.26 ± 0.03	0.22 ± 0.03	0.26 ± 0.02	0.22 ± 0.10
**Spinal Canal(C5)**	1.17 ± 0.15	0.62 ± 0.12	0.85 ± 0.13	0.54 ± 0.15

*Indicates that the value is less than 0.01.

## Discussion

Targeting α_v_β_6_-integrin with PET imaging has garnered great interest due to its specific upregulation in various malignancies. Consequently, a variety of α_v_β_6_-integrin targeted radiopharmaceuticals have been developed. Among these, [^68^Ga]Ga-Trivehexin, a trimer cyclopeptide combined with triazacyclononane-triphosphinate (TRAP), was proved to be clinically efficacious. A detailed exploration of manual in-house kit-like synthesis protocol was reported by Parul Thakral et al. However, considering the enormous potential value of [^68^Ga]Ga-Trivehexin in clinical applications and the lack of an automatically synthesis protocol, we designed to establish a module based automated synthesis method. This approach would expand the feasibility and reproducibility of [^68^Ga]Ga-Trivehexin production, avoid unnecessary contamination, and achieve clinical application requested purity and sterility. In this study, we detailed explored the effect of radiolabeling parameters on the iQS-TS synthesis module, including pH value and precursor loads, and successfully established a fully-automated synthesis protocol of [^68^Ga]Ga-Trivehexin.

Throughout the evaluation, the reaction temperature and reaction time were kept constant to achieve a homogenous and stable temperature distribution in the reaction vial. Unlike other peptide tracers, such as [^68^Ga]Ga-FAPI-46 (16) and [^68^Ga]Ga-PSMA-11 (17), the optimal RCY of [^68^Ga]Ga-Trivehexin was obtained under more acidic conditions, with pH values = 2.9 ~ 3.6. When pH<2.9, activity distribution in waste increased significantly, which suggests the hindrance of gallium-68 coordination possibly due to the excessive protonation of nitrogen atoms. Conversely, the decline in RCY under higher alkaline conditions mainly resulted in the losing activity on the C18 cartridge, as Ga(OH)_3_ colloid existed more dominantly (**Supplementary Figure S4**). Although good RCYs were achieved with high dose of precursor, considering economic cost and the injected dose of the cold peptide, we finally suggested 30 μg as the optimal amount.

To further evaluate the potential of clinical application of this protocol, all-sided and strict quality control was performed. Notably, the results of radiochemical purity, ethanol content, radionuclide purity and identity, pH value, bacterial endotoxins and sterility, stability all conformed to the requirements of the European Pharmacopoeia.

Distribution results from in-human PET imaging showed no associated non-specific uptake in major organs, which was consistent with the previous study (12). In the latest studies, due to the absence of any non-specific uptake in the background, [^68^Ga]Ga-Trivehexin was reported to exhibit better lesion delineation than [^18^F]F-FDG PET in PDAC cases expressing α_v_β_6_ integrin, as well as in patients with brain metastases (18, 19). In addition, the relatively low total body radiation dose (1.26E-02 mGy/MBq) allowed multiple [^68^Ga]Ga-Trivehexin PET scans, suggesting more conducive utilization for clinical diseases diagnosis. These results indicated the splendid potential of this [^68^Ga]Ga-Trivehexin automated synthesis protocol to be successfully applied to clinical imaging of α_v_β_6_-integrin profiling.

## Conclusion

In this study, a fully-automated synthesis protocol based on iQS-TS module was successfully established to obtain [^68^Ga]Ga-Trivehexin with a good radiochemistry yield, high radiochemical purity, reproducibility, and compliance with clinical requirement. The entire synthesis was performed using the module with a disposable cassette, greatly simplifying manual operation and avoiding unwanted radiation exposure. Thus, our automated protocol demonstrated a convenient and reliable synthesis of [^68^Ga]Ga-Trivehexin, contributing its broader practical clinical applications.

## Data availability statement

The original contributions presented in the study are included in the article/**Supplementary Material**, further inquiries can be directed to the corresponding authors.

## Ethics statement

The studies involving humans were approved by Medical Ethics Committee, Zhongnan Hospital of Wuhan University. The studies were conducted in accordance with the local legislation and institutional requirements. The participants provided their written informed consent to participate in this study. Written informed consent was obtained from the individual(s) for the publication of any potentially identifiable images or data included in this article.

## Author contributions

BW: Writing – original draft, Software, Methodology, Investigation, Formal analysis, Data curation. YJ: Writing – original draft, Investigation, Formal analysis, Data curation. JZ: Writing – review & editing, Methodology, Investigation. HW: Writing – review & editing, Formal analysis, Data curation. JW: Writing – review & editing, Resources. LL: Writing – review & editing, Resources. JH: Writing – review & editing, Resources, Project administration. ZX: Writing – review & editing, Validation, Supervision, Data curation. YH: Writing – review & editing, Validation, Project administration, Conceptualization.

## Supplementary material

Supplementary Figure 1 The structure of Trivehexin.

Supplementary Figure 2 The configuration of the cassette.

Supplementary Figure 3
**(A)** Radio-HPLC Chromatogram of [^68^Ga]Ga-Trivehexin; **(B)** UV-HPLC Chromatogram of [^68^Ga]Ga-Trivehexin.

Supplementary Figure 4 RCY and Radioactivity distribution on C18 cartridge at different pH values. RCY = radiochemical yield.
