# Supplementary figures and images for: Fully-automated production of [68Ga]Ga-Trivehexin for clinical application and its biodistribution in healthy volunteers
Source: Front Oncol. 2024 Aug 2;14:1445415. doi: 10.3389/fonc.2024.1445415 (PMC11327152; doi:10.3389/fonc.2024.1445415)

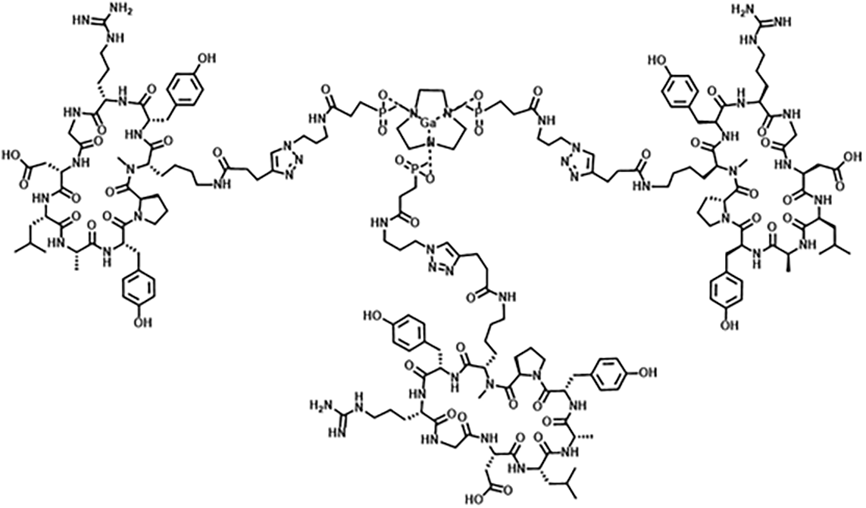

Supplement: Supplementary Figure 1 — The structure of Trivehexin. [file Image_1.tif]

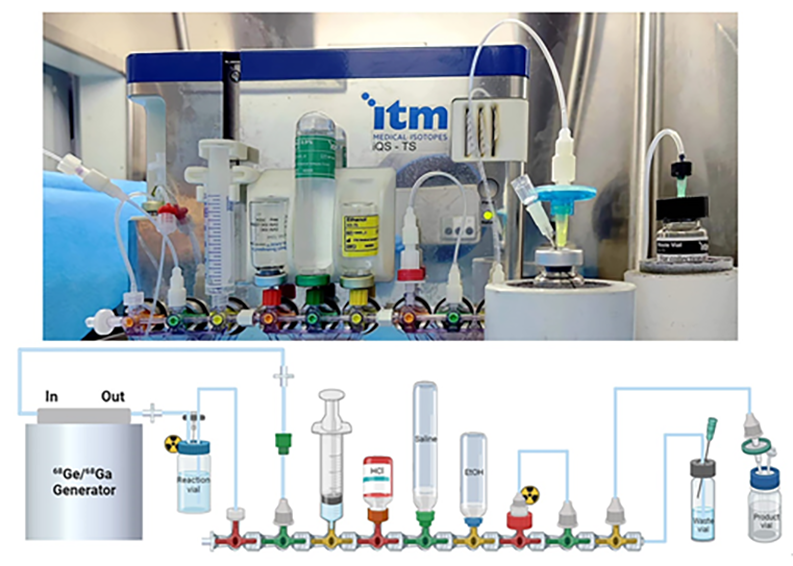

Supplement: Supplementary Figure 2 — The configuration of the cassette. [file Image_2.tif]

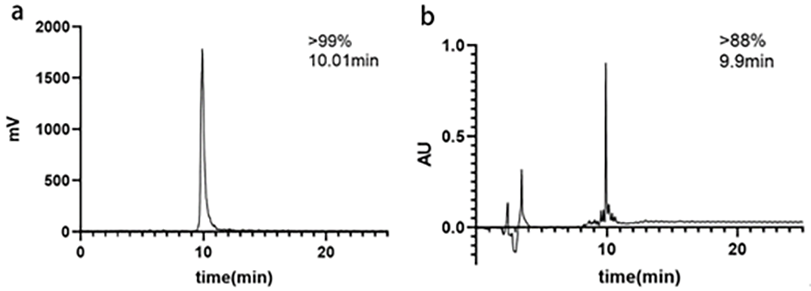

Supplement: Supplementary Figure 3 — (A) Radio-HPLC Chromatogram of [68Ga]Ga-Trivehexin; (B) UV-HPLC Chromatogram of [68Ga]Ga-Trivehexin. [file Image_3.tif]

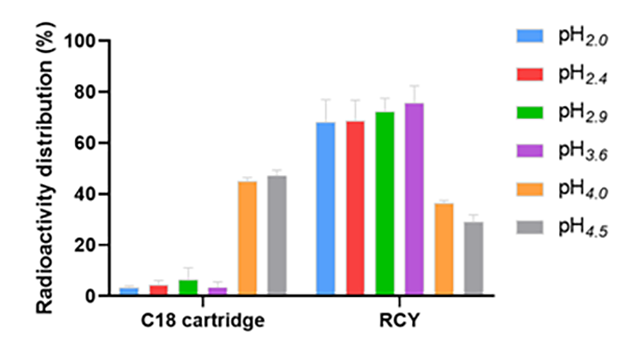

Supplement: Supplementary Figure 4 — RCY and Radioactivity distribution on C18 cartridge at different pH values. RCY = radiochemical yield. [file Image_4.tif]
