# Supplementary material for: Fully-automated production of [68Ga]Ga-Trivehexin for clinical application and its biodistribution in healthy volunteers
Source: Front Oncol. 2024 Aug 2;14:1445415. doi: 10.3389/fonc.2024.1445415 (PMC11327152; doi:10.3389/fonc.2024.1445415)
Supplement: Supplementary file 5 [file Table_1.docx]

**Table S1** Synthesis steps of iQS-Theranostics Synthesizer module.

| **Step** | **Procedure** | **Time (min)** |
| --- | --- | --- |
| 1 | Elution of the Generator | 2 |
| 2 | Labelling and RP cartridge conditioning | 10 |
| 3 | Purification and formulation | 3 |
| 4 | Filter integrity test is performed | 1 |
| 5 | Rinsing the cassette with residual saline | 4.5 |
| 6 | End of synthesis | / |
