# Supplementary material for: Fully-automated production of [68Ga]Ga-Trivehexin for clinical application and its biodistribution in healthy volunteers
Source: Front Oncol. 2024 Aug 2;14:1445415. doi: 10.3389/fonc.2024.1445415 (PMC11327152; doi:10.3389/fonc.2024.1445415)
Supplement: Supplementary file 6 [file Table_2.docx]

**Table S2** Clinical information of three healthy volunteers.

| **Number** | **Gender** | **Age (years)** | **Height (cm)** | **Weight (kg)** | **Injected Dose (MBq)** |
| --- | --- | --- | --- | --- | --- |
| 1 | Male | 48 | 172 | 65.9 | 122 |
| 2 | Female | 26 | 156 | 46.8 | 87 |
| 3 | Female | 29 | 165 | 61.2 | 113 |
