# Supplementary material for: Fully-automated production of [68Ga]Ga-Trivehexin for clinical application and its biodistribution in healthy volunteers
Source: Front Oncol. 2024 Aug 2;14:1445415. doi: 10.3389/fonc.2024.1445415 (PMC11327152; doi:10.3389/fonc.2024.1445415)
Supplement: Supplementary file 7 [file Table_3.docx]

**Table S3** Dose estimates for [^68^Ga]Ga-Trivehexin, calculated with OLINDA V2.2 based on the organ residence times

| **Organ** | **Dose(mGy/MBq)** |
| --- | --- |
| Adrenals | 3.22E-02 |
| Brain | 1.40E-03 |
| Esophagus | 1.98E-02 |
| Eyes | 4.25E-03 |
| Gallbladder Wall | 2.09E-02 |
| Left colon | 1.88E-02 |
| Small Intestine | 1.78E-02 |
| Stomach Wall | 1.77E-02 |
| Right colon | 1.86E-02 |
| Rectum | 1.96E-02 |
| Heart Wall | 1.73E-02 |
| Kidneys | 2.26E-01 |
| Liver | 1.58E-02 |
| Lungs | 8.72E-03 |
| Pancreas | 1.96E-02 |
| Prostate | 1.77E-02 |
| Salivary Glands | 1.53E-02 |
| Red Marrow | 1.16E-02 |
| Osteogenic Cells | 1.86E-02 |
| Spleen | 1.68E-02 |
| Testes | 1.41E-02 |
| Thymus | 1.31E-02 |
| Thyroid | 1.96E-02 |
| Urinary Bladder Wall | 8.24E-02 |
| **Total Body** | **1.26E-02** |
| **Effective Dose** | **1.67E-02** |
